# Fracture Resistance of Composite Fixed Partial Dentures Reinforced with Pre-impregnated and Non-impregnated Fibers

**DOI:** 10.5681/joddd.2012.003

**Published:** 2012-03-13

**Authors:** Ramin Mosharraf, Sepideh Torkan

**Affiliations:** ^1^Torabinejad Dental Research Center, Isfahan University of Medical Sciences, Isfahan, Iran; ^2^Associate Professor, Department of Prosthodontics, Faculty of Dentistry, Isfahan University of Medical Sciences, Isfahan, Iran; ^3^Post-graduate Student, Department of Orthodontics, Faculty of Dentistry, Shiraz University of Medical Sciences, Shiraz, Iran

**Keywords:** Fiber-reinforced composites, fracture resistance, non-impregnated fibers, pre-impregnated fibers

## Abstract

**Background and aims:**

The mechanical properties of fiber-reinforced composite fixed partial dentures (FPDs) are af-fected by fiber impregnation. The aim of this in vitro study was to compare the fracture resistance of composite fixed partialdentures reinforced with pre-impregnated and non-impregnated fibers.

**Materials and methods:**

Groups (n=5) of three-unit fiber-reinforced composite FPDs (23 mm in length) from maxillary second premolar to maxillary second molar were fabricated on two abutments with pontic width of 12 mm. One group was fabricated as the control group with composite (Gradia) and the other two groups were fabricated with composite (Gradia) reinforced with pre-impregnated fiber (Fibrex ribbon) and non-impregnated fiber (Fiber braid), respectively. The specimens were stored in distilled water for one week at 37°C and then tested in a universal testing machine by means of a three-point bending test. Statistical analysis consisted of one-way ANOVA and a post hoc Scheffé’s test for the test groups (α=0.05).

**Results:**

Fracture resistance (N) differed significantly between the control group and the other two groups (P<0.001), but there were no statistically significant differences between the pre-impregnated and non-impregnated groups (P=0.565). The degree of deflection measured (mm) did not differ significantly between the three groups (P=0.397), yet the mean deflection measured in pre-impregnated group was twice as that in the other two groups.

**Conclusion:**

Reinforcement of composite with fiber might considerably increase the fracture resistance of FPDs; how-ever, the type of the fiber used resulted in no significant difference in fracture resistance of FPD specimens.

## Introduction

Fiber-reinforced composites (FRCs) have been increasingly studied over the past three decades since they offer a promising approach.^1 - 3^ Brown^4^ has discussed the current dental applications of fiber reinforcement, including dental cements and splints, fibers made into structures for use in direct and indirect composites restorations and denture bases.

Fiber-reinforced composite fixed partial dentures (FPDs) are an alternative to metal-ceramic adhesive FPDs.^5 , 6^ They have been extensively studied during recent years since they give restorations a considerable strength. Such restorations resist high levels of mechanical stress, have a low weight, are esthetically satisfactory, can be produced easily and are cost-effective.^7 - 12^

Although much is known about the properties of FRC itself and it has been reported that reinforcement with fiber is an effective method for a considerable improvement in flexural properties of indirect composite-resin restorations,^13 - 15^ less information is available on the properties of a material combination of FRC, especially when used for reinforcement of restorative composite resin.^16^

Fiber reinforcement is only successful if the loading force can be transferred from the matrix to the fiber. Mechanical properties of FRCs are influenced in many ways, and the factors affecting their strength include position of fibers, quantity of fibers, impregnation of fibers with the polymer matrix, adhesion of fibers to the polymer matrix, properties of fibers, properties of polymer matrix, and water absorption of the FRC matrix.^13^

Many authors have investigated the impregnation of fibers with the matrix because poor impregnation creates problems with the use of FRC in dentistry.^1, 17 - 22^, If there are regions in which the fibers are not completely embedded in resin, there will be voids that increase water sorption and, thus, decrease mechanical properties of the FRC.^7 , 17 , 18^

Poorly impregnated fibers cause another problem: the increase in water sorption in FRC,^23 , 24^ which compromises mechanical properties.^25 , 26^ Voids and cracks in the laminate allow water to enter. A reliable adhesion between the fibers and the matrix reduces voids and cracks, which can limit water sorption. To solve all these problems, pre-impregnated FRC are used. It has been concluded that the highest fracture resistance could be seen in FPDs reinforced with pre-impregnated fibers.^27^The aim of this in vitro study was to compare the fracture resistance of composite fixed partial dentures reinforced with pre-impregnated and non-impregnated fibers. The null hypothesis was that reinforcement with fiber and type of fiber impregnation would not affect the primary fracture resistance.

## Materials and Methods

**Figure 1:**
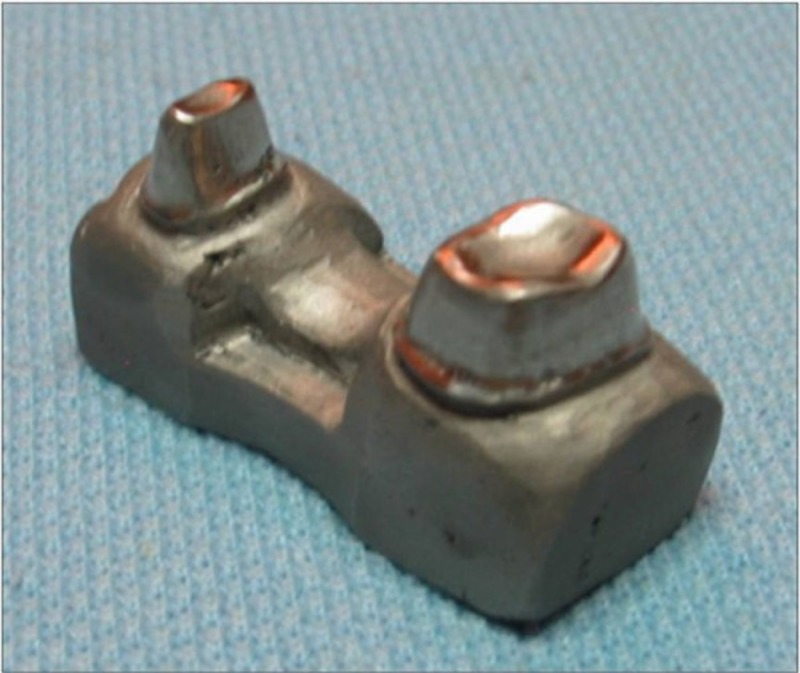
Prepared metallic teeth.

Three groups of three-unit fixed partial dentures (n=5/group) were prepared from GC Gradia composite (Gradia; GC Corp, Tokyo, Japan) with or without fiber reinforcement and tested to failure. Acrylic resin teeth were used for the fabrication of the FPDs. The mesial abutment tooth was a prepared maxillary premolar (height: 5 mm), while a prepared maxillary molar (height: 5 mm) represented the distal abutment. The acrylic resin teeth were recessed so that the finish line was a circular shoulder (1 mm) with rounded internal axiogingival line angles. The buccolingual and mesiodistal convergence angle was 10 degrees. Tooth diameter at the internal line angle of the circular shoulder was 3.5 mm (mesiodistal) and 6.5 mm (buccolingual) for the premolar. Tooth diameters for the molar were 6 mm and 7.5 mm, respectively. Then the prepared teeth were secured in a wax pattern in the required distance of 12 mm. Because fiber reinforced fixed partial dentures generally have been used for replacing a single premolar or molar with an intra-abutment span not exceeding 15 mm.^17^ With this method, a standard cast for a three unit FPD was designed. The model was then cast with metal (Nickel-chrome alloy; Wiron 88; Bego, Bremen, Germany) (Figure 1). Composite resin (Gradia; GC Corp) was used to form a three-unit FPD on the metal cast. Pontic height at the point where it met the connectors was 4.3 mm. A silicone mold was fabricated from the prepared FPD and was used to reproduce 15 master casts, which were poured with a type III dental stone (Moldano; Heraeus Kulzer, Dormagen, Germany). A Vacuform pull-down matrix was prepared on each cast to better standardize the exterior outer form of the FPDs. This way it was ensured that the FPDs demonstrated equal dimensions. By using the same pull-down, the outer forms of the FPDs were also duplicated in an improved standardized fashion.

### Control Group

There was no fiber reinforcement in this group. Gradia composite was adapted to the two abutment dies; the pull-down was also filled with composite and was gently placed on the metal model. The composite was light-cured (Monitex ‘Bluex, GT1200’, Monitex Industrial Co., Taiwan) for 20 seconds from each side through the clear matrix. The matrix was removed and then the fixed partial denture was removed from the dies and trimmed using fine tungsten carbide points to clearly define marginal fit and inter-proximal contact areas.

###  Pre-impregnated Group

Construction of the fixed partial dentures in this group was similar to that in the control group except as follows: composite resin was placed between the abutments up to the occlusal surface and cured for 20 seconds. Then a 1-mm wide reinforcement ribbon (Fibrex. Ribbon, Angelus Dental Solution, Lonndrina, Brazil) was cut to the required length to span across the pontic area and to overlay across the central fossae of the copings. The ribbon was light-cured for 20 seconds along its entire length.

### Non-impregnated Group

Construction of the fixed partial dentures in this group was similar to that in the pre-impregnated group except as follows: The reinforcement ribbon (Fiber-braid; NSI Dental PTY, Hornsby, Australia) was carefully impregnated with composite primer (Composite Primer_; _GC Corp). When the ribbon became transparent in appearance, indicating saturation by unfilled resin, it was gently placed over the abutments the same way as that in group B.

The fixed partial dentures were polymerized off the dies for 20 minutes in a Gradia laboratory light-curing unit (Labolight; GC Corp). The fixed partial dentures of all the groups were stored in distilled water at 37°C for 7 days. One hour after removal of the specimens from the incubator (to allow the specimens to return to room temperature) they were tested dry at room temperature. Before testing the fixed partial dentures were cemented using non-eugenol provisional cement (Temp Bond NE, Kerr, Karlsruhe, Germany) to the metallic support that was designed for this test.

The FPDs were subjected to a static three-point load test until fracture with a universal testing machine (TLCLO, Dartec series, England) according to ISO: 10477.

**Figure 2:**
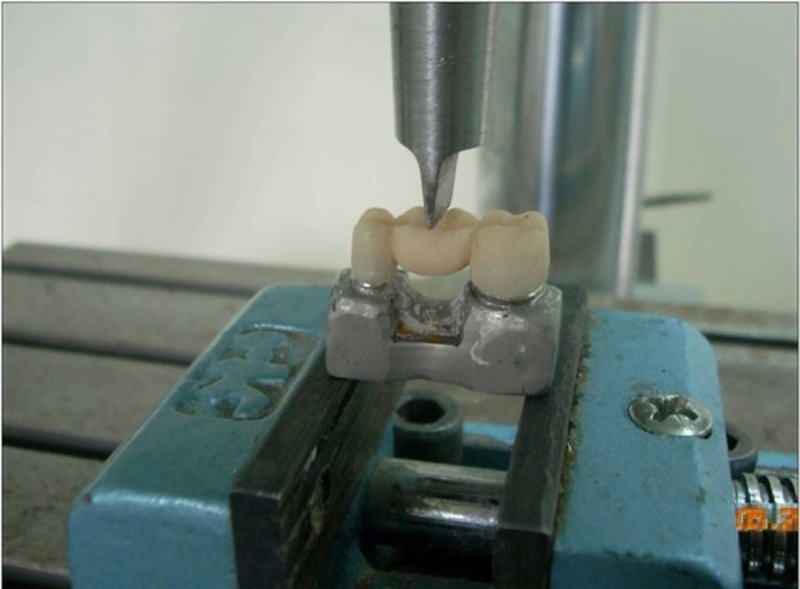
Specimen in universal testing machine.

Force was applied perpendicular to the center of the FPD. The center was marked at the midpoint of the pontic width. The load was applied to the FPDs by a steel ram placed in the central fossa (Figure 2).

The test specimens were continuously loaded at a crosshead speed of 1 mm/min. The beginning of the specimen damage was classified as the initial failure (IF). IF was denoted if at least two of the following conditions were present: 1) a sharp decline in the load/displacement curve, called a knee or corner; 2) visible signs of fracture; 3) audible emissions, caused by the generation of elastic waves by crack formation and/or progression.^28^ The maximum force (N) and degree of deflection at fracture time was recorded by universal testing machine.

Statistical analysis of the results was carried out with one-way ANOVA and a post hoc Scheffé’s tests (α=0.05).

## Results

For each specimen the data recorded included the force measured at the time of the primary fracture (N) and the degree of deflection of the specimens at the time of fracture (mm). One-way ANOVA revealed a significant difference between the control group and the other two groups in primary fracture (P<0.001). A post hoc Scheffé’s test showed a significant difference between the control group and the two other groups (P<0.001), but there were no significant differences between the pre-impregnated and non-impregnated groups in their primary fracture resistance (P=0.565) (Table 1).

**Table 1:** Descriptive data of the study

	Control		Non-impregnated		Pre-impregnated	
	Mean	SD	Mean	SD	Mean	SD
Primaryfracture force (N)	67.2	2.06	105	4.57	102	22.57
Deflection (mm)	0.134	0.04	0.114	0.11	0.242	0.13

Regarding deflection, one-way ANOVA revealed no significant differences between the three groups (P=0.397); however, the mean deflection measured in pre-impregnated group was twice as much as that in the other two groups.

## Discussion

This in vitro study examined the fracture resistance of three-unit composite fixed partial dentures reinforced with pre-impregnated and non-impregnated fibers and compared them with non-reinforced specimens. The fracture resistance of pre-impregnated and non-impregnated groups were significantly higher than that in the control group (P<0.001), consistent with the results of other studies, ^6 , 7 , 13 , 27^ which have demonstrated that use of reinforcing fibers improve the flexural strength of composite resins in comparison with the unreinforced control specimens.^13 - 15^

Pfeiffer ^27^ showed that the results achieved with impregnated fibers (Vectris) were better than those achieved with non-impregnated fibers (Ribbond). However, in the present study, the results achieved with impregnated fibers were the same as those with non-impregnated fibers (P=0.565). This finding could be explained by the specimens being in the form of a conventional three-unit FPD that have higher composite volume (or lower fiber volume fraction) than usual standard bar type specimens in other studies;^29^ in addition, it might have be explained by the unique composition of Gradia composite, which is claimed to be a micro-ceramic composite by the manufacturing company. It has been demonstrated that the composition of the overlying veneering composite plays a critical role in the flexural properties of the final fiber-reinforced restoration.^29^

In addition, the adhesive resin (the coupling agent) used to impregnate the non-impregnated fibers before reinforcement might also have a significant effect on the flexural strength of FRCs.^21 , 22 , 27^Therefore, the high values measured in the non-impregnated group might be related to the compatibility of the type of resin used for impregnating the fibers with the type of the composite resin used in the present study. Therefore, reinforcement of fiber systems should be carried out with carefully determined and defined fiber concentration and coordinated material combination, recommended by the manufacturer.^27^

The full coverage design used in this investigation might have affected the results because the retainer design seems to play an important role in the efficacy of reinforcement. However, it seems that more research is needed to determine whether reinforcing these retainers, especially in three-unit fixed partial dentures, is a must or not.

Although the values measured for deflection in the control and non-impregnated groups were lower than those in the pre-impregnated group, from the statistical point of view, there were no significant differences between the groups (P=0.397). The higher deflection values in the pre-impregnated group could be a result of the type, orientation and quantity of the fibers used. On the other hand, in the present study the specimens were rigidly anchored, which is different from that in human jaws. It has been reported that the support abutments that do not move during bending result in the deflection being limited and the fracture resistance values measured in this type of investigations are generally higher than those determined with supports that allow their movement.^27 , 30^

Concerning the deflection test, pre-impregnated specimens seem to be more flexible since the specimens in this group showed a little more deflection before fracture occurred. This result could be of great importance in cases in which more flexibility is required, such as patients with parafunctional habits (bruxism, clenching) and in dental implants in which the cushioning effect of the material implied seems more appropriate.^27^

In this in vitro study, axial forces were applied to the center of the occlusal pontic area. Clinically, besides axial forces, lateral forces and fatigue loading on fiber-reinforced composite FPDs should be considered. These forces might have an additional effect on the mechanical properties of FPDs.^27^

One limitation of this study was the non-inclusion of an artificial aging process, such as thermocycling and dynamic loading, which would have simulated this negative effect on fracture resistance.

## Conclusion

In summary, it can be concluded that the load to fracture of the type of composite resins used in the present study significantly increased by adding fiber reinforcing frameworks but there were no significant differences between impregnated and non-impregnated fiber reinforced FPDs.

## References

[1] ferilich ma, karmaker ac, burston cj, goldberg aj (1998). development and clinical applications of a light-polymerized fiber-reinforced composite. j prosthet dent.

[2] ruyter ie, ekstrand k, bjork n (1986). development of carbon/graphite fiber reinforced poly (methyl methacrylate) suitable for implant-fixed dental bridges. dent mater.

[3] bjork n, ekstrand k, ruyter re (1986). implant-fixed, dental bridges from carbon/graphite fibre reinforced poly (methyl methacrylate). biomaterials.

[4] brown d (2000). fiber-reinforced materials. dent update.

[5] butterworth c, ellakwa ae, shortall a (2003). fibre-reinforced composites in restorative dentistry. dent update.

[6] vallittu pk, sevelius c (2000). resin-bonded, glass fiber-reinforced composite fixed partial dentures: a clinical study. j prosthet dent.

[7] behr m, rosentritt m, leibrock a, schneider-feyrer s, handel g (1999). in-vitro study of fracture strength and marginal adaptation of fibre-reinforced adhesive fixed partial inlay dentures. j dent.

[8] ramos v jr, runyan da, christensen lc (1996). the effect of plasma-treated polyethylene fiber on fracture strength of polymethyl methacrylate. j prosthet dent.

[9] vallittu pk (1999). flexural properties of acrylic resin polymers reinforced with unidirectional and woven glass fibers. j prosthet dent.

[10] kim sh, watts dc (2004). effect of glass-fiber reinforcement and water storage on fracture toughness (kic) of polymer-based provisional crown and fpd materials. int j prosthodont.

[11] kangasniemi i, vallittu p, meiers j, dyer sr, rosentritt m (2003). consensus statement on fiber-reinforced polymers: current status, future directions, and how they can be used to enhance dental care. int j prosthodont.

[12] vallittu pk (2004). survival rates of resin-bonded, glass fiber-reinforced composite fixed partial dentures with a mean follow-up of 42 months: a pilot study. j prosthet dent.

[13] al-darwish m, hurley rk, drummond jl (2007). flexure strength evaluation of a laboratory-processed fiber-reinforced composite resin. j prosthet dent.

[14] ellakwa ae, shortall ac, shehata mk, marquis pm (2001). the influence of fibre placement and position on the efficiency of reinforcement of fibre reinforced composite bridgework. j oral rehabil.

[15] ellakwa a, shortall ac, marquis p (2003). influence of fibre position on the flexural properties and strain energy of a fibre-reinforced composite. j oral rehabil.

[16] mallick pk (1993). fiber-reinforced composites. materials, manufacturing, and designs.

[17] song hy, yi yj, cho lr, park dy (2003). effects of two preparation designs and pontic distance on bending and fracture strength of fiber-reinforced composite inlay fixed partial dentures. j prosthet dent.

[18] vallittu pk, ruyter ie, ekstrand k (1998). effect of water storage on the flexural properties of e-glass and silica fiber acrylic resin composite. int j prosthodont.

[19] goldberg aj, burstone cj (1992). the use of continuous fiber reinforcement in dentistry. dent mater.

[20] vallittu pk (1998). some aspects of the tensile strength of unidirectional glass fibre-polymethyl methacrylate composite used in dentures. j oral rehabil.

[21] ellakwa ae, shortall ac, shehata mk, marquis pm (2002). influence of bonding agent composition on flexural properties of an ultra-high molecular weight polyethylene fiber-reinforced composite. oper dent.

[22] ellakwa ae, shortall ac, marquis pm (2002). influence of fiber type and wetting agent on the flexural properties of an indirect fiber reinforced composite. j prosthet dent.

[23] miettinen va, narva kk, vallittu pk (1999). water sorption, solubility and effect of post-curing of glass fibre reinforced polymers. biomaterials.

[24] ladizesky nh, chow tw (1992). the effect of interface adhesion, water immersion and anatomical notches on the mechanical properties of denture base resins reinforced with continuous high performance polyethylene fibers. aust dent j.

[25] soderholm kj, roberts mj (1990). influence of water exposure on the tensile strength of composites. j dent res.

[26] soderholm kj, zigan m, ragan m, fischlschweiger w, bergman m (1984). hydrolytic degradation of dental composites. j dent res.

[27] pfeiffer p, grube l (2003). in vitro resistance of reinforced interim fixed partial dentures. j prosthet dent.

[28] dyer sr, sorensen ja, lassila vl, vallittu pk (2005). damage mechanics and load failure of fiber-reinforced composite fixed partial dentures. dent mater.

[29] ellakwa a, shortall a, shehata m, marquis p (2001). influence of veneering composite composition on the efficacy of fiber-reinforced restorations (frr). oper dent.

[30] kayacan r, ballarini r, mullen rl (1997). theoretical study of the effects of tooth and implant mobility differences on occlusal force transmission in tooth/implant-supported partial prostheses. j prosthet dent.

